# Ex-vivo quantification of ovine pia arachnoid complex biomechanical properties under uniaxial tension

**DOI:** 10.1186/s12987-020-00229-w

**Published:** 2020-11-12

**Authors:** Gabryel Conley Natividad, Sophia K. Theodossiou, Nathan R. Schiele, Gordon K. Murdoch, Alkiviadis Tsamis, Bertrand Tanner, Gabriel Potirniche, Martin Mortazavi, David A. Vorp, Bryn A. Martin

**Affiliations:** 1 Department of Chemical and Biological Engineering, University of Idaho, 875 Perimeter Dr. MS0904, Moscow, ID 83844 USA; 2 Department of Animal & Veterinary Science, University of Idaho, 875 Perimeter Dr. MC1122, Moscow, ID 83844-2330 USA; 3 School of Engineering, University of Leicester, University Road, Leicester, LE1 7RH UK; 4 Department of Integrated Physiology and Neuroscience, Washington State University, 1815 Ferdinand’s Lane, Pullman, WA 99164 USA; 5 Department of Mechanical Engineering, University of Idaho, 875 Perimeter Dr. MC1122, Moscow, ID 83844-1122 USA; 6 National Skull Base Foundation, 2100 Lynn Rd #120, Thousand Oaks, CA 91360 USA; 7 Departments of Bioengineering, Cardiothoracic Surgery, Surgery, and Chemical & Petroleum Engineering, and the Clinical and Translational Sciences Institute, University of Pittsburgh, 3700 O’Hara Street, Pittsburgh, PA 15261 USA; 8 Alcyone Therapeutics Inc, Lowell, MA 01852 USA

## Abstract

**Background:**

The pia arachnoid complex (PAC) is a cerebrospinal fluid-filled tissue conglomerate that surrounds the brain and spinal cord. Pia mater adheres directly to the surface of the brain while the arachnoid mater adheres to the deep surface of the dura mater. Collagen fibers, known as subarachnoid trabeculae (SAT) fibers, and microvascular structure lie intermediately to the pia and arachnoid meninges. Due to its structural role, alterations to the biomechanical properties of the PAC may change surface stress loading in traumatic brain injury (TBI) caused by sub-concussive hits. The aim of this study was to quantify the mechanical and morphological properties of ovine PAC.

**Methods:**

Ovine brain samples (n = 10) were removed from the skull and tissue was harvested within 30 min post-mortem. To access the PAC, ovine skulls were split medially from the occipital region down the nasal bone on the superior and inferior aspects of the skull. A template was used to remove arachnoid samples from the left and right sides of the frontal and occipital regions of the brain. 10 ex-vivo samples were tested with uniaxial tension at 2 mm s^−1^, average strain rate of 0.59 s^−1^, until failure at < 5 h post extraction. The force and displacement data were acquired at 100 Hz. PAC tissue collagen fiber microstructure was characterized using second-harmonic generation (SHG) imaging on a subset of n = 4 stained tissue samples. To differentiate transverse blood vessels from SAT by visualization of cell nuclei and endothelial cells, samples were stained with DAPI and anti-von Willebrand Factor, respectively. The Mooney-Rivlin model for average stress–strain curve fit was used to model PAC material properties.

**Results:**

The elastic modulus, ultimate stress, and ultimate strain were found to be 7.7 ± 3.0, 2.7 ± 0.76 MPa, and 0.60 ± 0.13, respectively. No statistical significance was found across brain dissection locations in terms of biomechanical properties. SHG images were post-processed to obtain average SAT fiber intersection density, concentration, porosity, tortuosity, segment length, orientation, radial counts, and diameter as 0.23, 26.14, 73.86%, 1.07 ± 0.28, 17.33 ± 15.25 µm, 84.66 ± 49.18°, 8.15%, 3.46 ± 1.62 µm, respectively.

**Conclusion:**

For the sizes, strain, and strain rates tested, our results suggest that ovine PAC mechanical behavior is isotropic, and that the Mooney-Rivlin model is an appropriate curve-fitting constitutive equation for obtaining material parameters of PAC tissues.

## Introduction

**Fig. 1:**
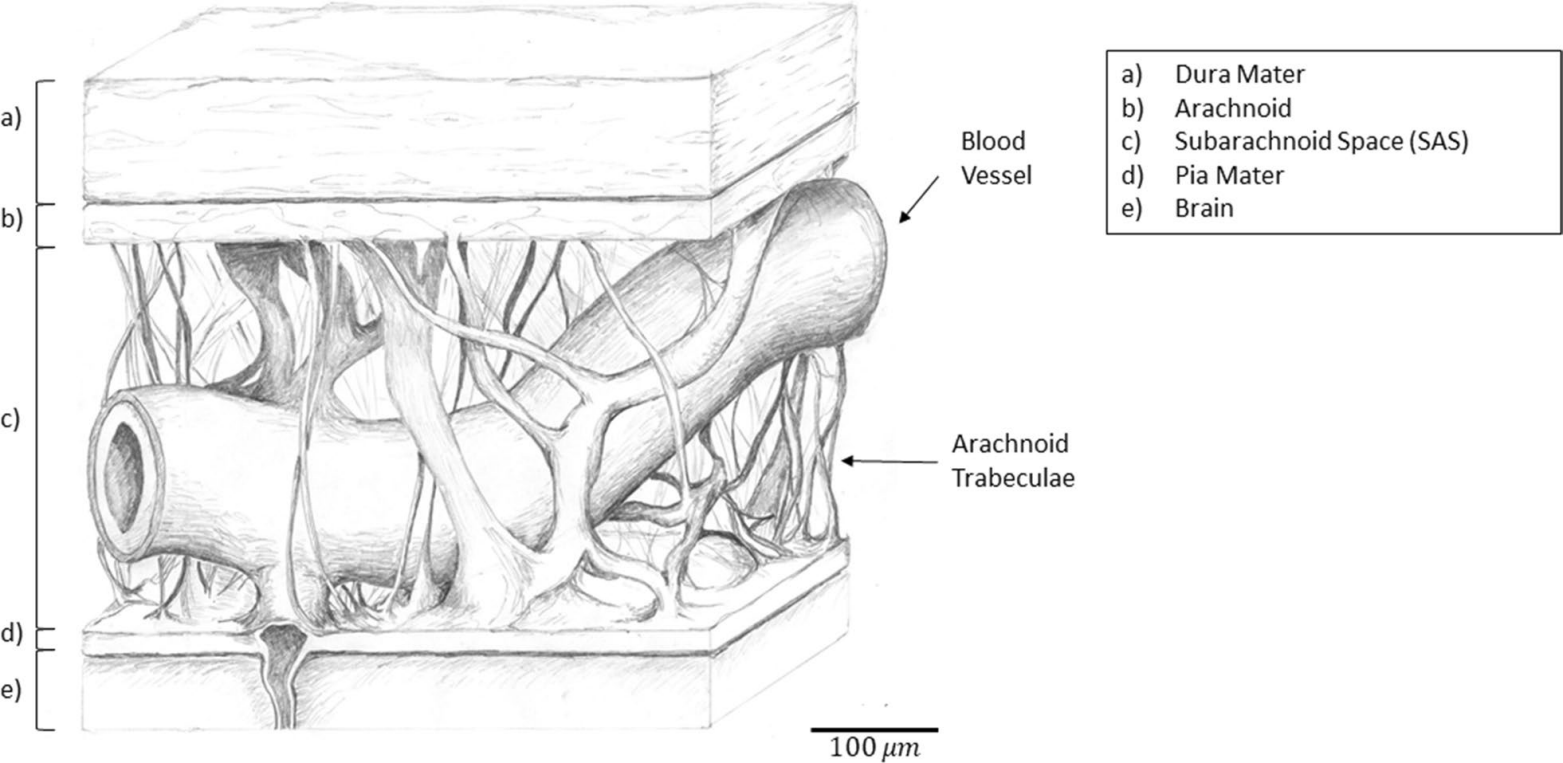
Anatomical drawing of pia arachnoid complex showing the **a** dura mater, **b** arachnoid mater, **c** subarachnoid space containing the arachnoid trabeculae fibers and blood vessels, **d** pia mater, and **e** brain tissue

The pia-arachnoid complex (PAC), commonly known as the leptomeninges of the brain, provides mechanical stability for the brain and spinal cord in all vertebrate species [1, 2]. The PAC lies deep to the dura mater and superficial to the brain, and is comprised of the pia mater, arachnoid mater and the subarachnoid space (SAS). The SAS is filled with cerebrospinal fluid (CSF) and contains subarachnoid trabeculae (SAT) which span the intermediate area of the meninges (Fig. 1). SAT are collagen fibers that develop as columns or sheets spanning the SAS, with lateral and transverse orientations relative to the pia and arachnoid mater [1, 3]. The unique PAC morphology suggests that one of its potential functions is to protect the brain from traumatic brain injury (TBI) through energy distribution and absorption [4].

Existing studies have identified the significance of the physiological and biomechanical properties of PAC in central nervous system (CNS) pathologies, such as TBI, spinal canal stenosis, and Space Flight Associated Neuro-Ocular syndrome (also called SANS), as well as in applications such as CSF-based drug delivery [4–7]. Neurosurgical dissection of spinal arachnoiditis, or inflammation of the PAC surrounding the spinal cord, is a procedure believed to reduce CSF flow obstruction by removing SAT fibers [7–12]. Gottschalk et al. found *in-vivo* flow alterations due to spinal arachnoiditis [13]. The relevance of SAT fibers has also been investigated in the context of their potential role in CSF drug delivery and solute transport [14–16]. Additionally, Killer et al. showed a potential for increased intraocular pressure caused by SAT fibers and by extension this could potentially affect astronauts with space flight associated neuro-ocular syndrome [17]. Taken together, available data implicates the PAC and SAT in CNS pathologies. Further investigations of the PAC are needed to improve both treatment options and preventative care.

Increased knowledge of PAC biomechanical properties may improve understanding of CNS pathology following trauma and enhance potential post-trauma treatments. Currently, a limited understanding of the mechanical behavior of the PAC and SAT hinders the development of accurate pathophysiological models of the CNS. A common method of modeling mechanical responses in the cranial cavity during TBI is finite element analysis. In order to increase model accuracy, biomechanical properties and microanatomy of the PAC need to be quantified. Previous studies have excluded or used assumed PAC biomechanical properties in computational fluid dynamic-models to investigate CSF dynamics [14, 15, 18]. SAT fibers have been found to potentially increase CSF mixing and pressure gradients throughout the CNS [14, 16, 19], and prior studies have quantified bovine PAC properties under uniaxial tension, traction, and shear [20–22]. However, more data on PAC across species are needed to show inter-species differentiation and elucidate how PAC biomechanical properties may affect transient stress loading on the brain during TBI.

The objective of the present study was to quantify nonlinear elastic properties under uniaxial tension and derive the appropriate material parameters of fresh ovine PAC, using the Mooney-Rivlin curve-fit model. Additionally, we aimed to characterize SAT structure and orientation through second-harmonic generation (SHG) imaging, with an automated collagen fiber structural analysis. Results of this study improve the understanding of the structure and biomechanical function of the PAC across species, which will advance finite element models and potentially lead to improved understanding and treatment of PAC-related pathologies.

## Methods

### PAC tissue collection

**Fig. 2:**
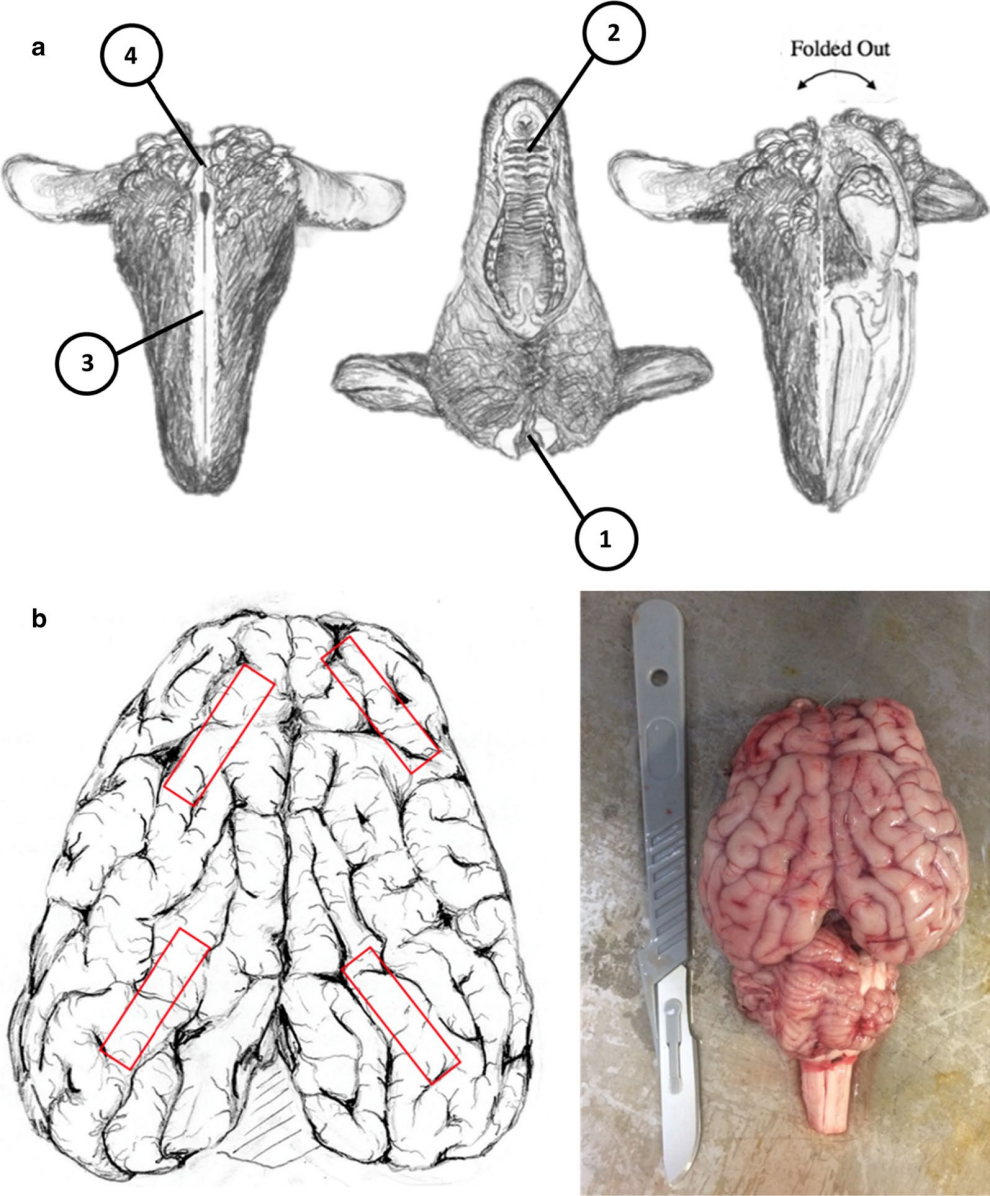
Procedure for dissection of the ovine PAC tissue. **a** Initial midline incision is made on superior and inferior aspect of the skull with lower mandible removes. Cuts are made sequentially (1–4). **b** Location of dissection of occipital and frontal PAC tissue sample removal and visual image of dissected ovine brain

Ten ovine brains from approximately 1-year-old animals were harvested from USDA inspected sheep at the University of Idaho Vandal Meats facility. The animals were euthanized using a captive bolt and immediately exsanguinated and decapitated. The captive bolt was applied on the forehead, slightly above a line drawn between the eyes. All samples were visually inspected for brain tissue damage and prepared for brain removal at < 10 min (min) post-mortem. Brain samples were extracted within 30 min post-mortem to minimize potential PAC breakdown. To access the PAC, the lower mandible was removed by making clean proximal cuts through the mastoid muscle, connective tissues, and temporomandibular joints on either side of the mouth (Fig. 2a). A strip of skin ~ 6 cm wide was removed from the medial portion of the skull to expose a clear strip of bone to split. The skull was then flipped upside down to expose the inferior portion of the skull. A steel wedge and hammer were used to split through the hard and soft palate of the mouth. The head was flipped again and split from the nasal bone along the exposed strip of bone to the superior occipital region. The skull was flipped upside down again to split the inferior portion of the occipital region, and all connective tissue was cut. During this procedure, the skull was examined to ensure that the PAC tissue remained intact. The skull was then separated carefully on the medial cut and the brain was removed in its entirety.

The brain was kept moist post-harvest with artificial CSF (EcoCyte Bioscience, Austin, Texas USA) while four samples were removed from the left and right occipital and frontal lobes (Fig. 2b). Each sample was measured with a clear rectangular template measuring 25 mm long, 10 mm wide, and 3 mm thick. A sample thickness of approximately 3 mm was chosen to include the PAC layer and underlying brain tissue. Extraction locations varied slightly so that each specimen would contain at least one sulcus or gyrus, and to avoid any obvious physical damage from the captive bolt traumatic injury site. Samples were visually inspected for tissue damage and either submerged in artificial CSF for biomechanical testing or fixed in 4% paraformaldehyde overnight for imaging. Fixed samples were washed 3 times for 5 min each in phosphate buffered saline (PBS) and stored in PBS at 4 °C and imaged at a later time. Biomechanical tests were performed within 5 h post-mortem, using samples submerged in artificial CSF immediately following dissection.

### PAC biomechanical testing preparation

**Fig. 3:**
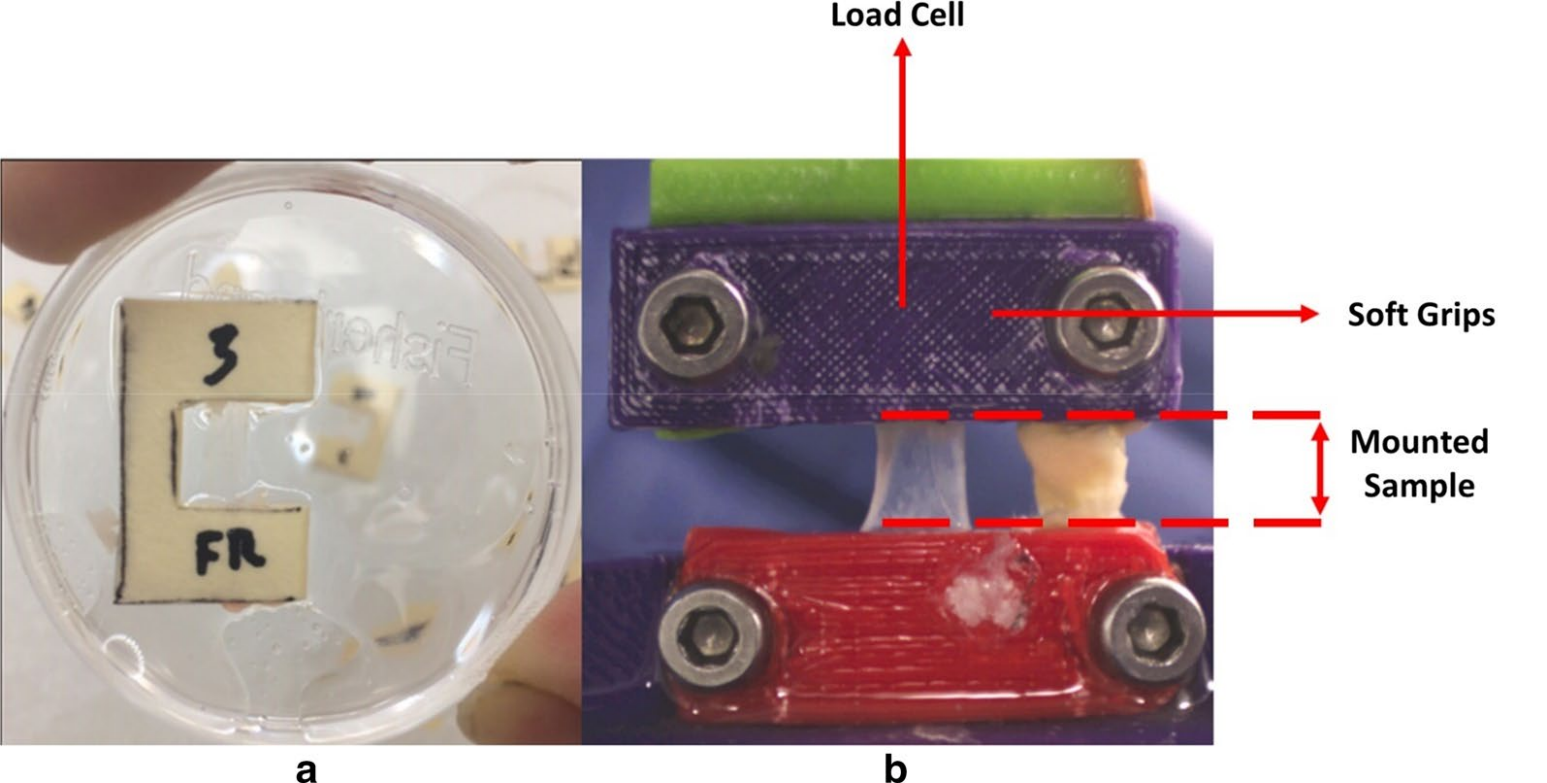
**a** PAC with brain matter removed and placed in C-shaped template used for fixture into the load cell. **b** Bioreactor apparatus with mounted sample within grips and orientation of load cell unixial tension

Immediately prior to biomechanical testing, individual brain samples were placed in a 49-mm diameter petri dish with the arachnoid mater facing down and continually moistened with artificial CSF. Room temperature (22 °C) was maintained throughout uniaxial tension tests. Each sample cut to ~ 15 mm long × 5 mm wide and placed on a c-shaped template with a 10-mm long window (Fig. 3a). Each end was secured in this c-clamp using cyanoacrylate glue. The soft brain matter was gently teased away using a scalpel and flat-head forceps until the translucent PAC was isolated. The dissected tissue was visually confirmed to be the PAC using a dissecting microscope, and tissue integrity was assessed before the sample was considered testable.

### PAC thickness measurement

To avoid potentially damaging testable PAC samples, a separate set of brain samples (n = 10) were used for an average PAC thickness measurement. A caliper (Model 293-340-30 Mitutoyo, Japan), equipped with a ratchet thimble, was used to measure the thickness of each PAC sample three times to obtain an average sample thickness. The sample was gently placed flat on the lower caliper clamp. The caliper was slowly tightened until the rachet thimble turned over and a visual examination showed the caliper clamps were in full contact of both side of the PAC. The average PAC thickness was calculated and used for subsequent calculations related to biomechanical tests.

### Uniaxial tensile testing

A custom small-scale uniaxial tensile load frame with a 150 g maximum capacity load cell (Model 31, Honeywell, Columbus, OH) was used to evaluate biomechanical properties of each PAC sample [23]. PAC samples were mounted and secured into custom soft tissue grips, and the paper c-clamp frame was cut to ensure that only the PAC tissue was loaded. Once mounted, width and length of the sample were measured using ImageJ (NIH, Bethesda, MD). PAC samples were pulled-to-failure in tension at a constant rate of 2 mm s^−1^ (Fig. 3b) while a LabVIEW program (National Instruments, Austin TX) recorded force and displacement data at 100 Hz. A total of 22 ovine PAC samples were tested under uniaxial tension from the frontal and occipital lobes. Samples were discarded if they failed at the grip (n = 6), a large blood vessel was present within the sample (n = 2), the cross-section was non-uniform (n = 1), or the increasing stress–strain curve was non-monotonic (n = 3). All 10 samples included in the analysis failed at the mid-substance and had a uniform cross-section.

### Mooney-Rivlin curve fitting and material parameter estimation

**Fig. 4:**
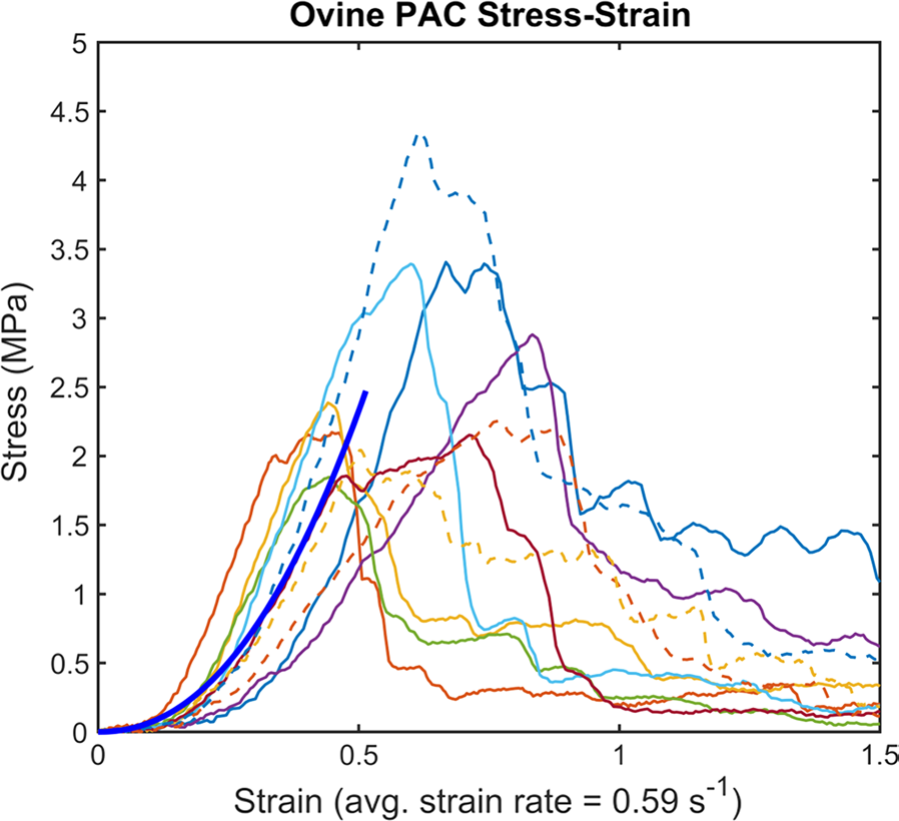
Stress–strain curves of the ovine PAC specimens when subjected to uniaxial tension. Average curve shown in dark blue

The force–displacement data collected from the experiments were analyzed using a custom MATLAB script (MathWorks, Natick, MA). A Mooney-Rivlin curve fit model (Eq. 1) for uniaxial tension was used to fit the obtained average stress–strain curve (Fig. 4), with stretch ratio $\lambda =\varepsilon +1$. Mooney-Rivlin constants,${C}_{10},{C}_{01 },{C}_{20}$, were estimated by a nonlinear least-squares fit using the MATLAB curve fit tool (Vers. R2018B).

1 $${\sigma }_{Uniaxial}^{Eng}=2{C}_{10}\left(\lambda -\frac{1}{{\lambda }^{2}}\right)+2{C}_{01}\left(\lambda -\frac{1}{{\lambda }^{3}}\right)+4{C}_{20}\left(\lambda -\frac{1}{{\lambda }^{2}}\right)\left({\lambda }^{2}+\frac{2}{\lambda }-3\right)$$

Young’s modulus (E) was calculated by tensile stress ($\sigma$) over engineering extensional strain ($\varepsilon$) over the linear region (Eq. 2). Tensile stress ($\sigma$) is the force exerted on the sample F divided by the cross-sectional area of the sample A. Engineering strain (ε) is the change in sample length ΔL divided by the initial length L_0_ of the sample (Eq. 3). The strain rate was calculated as the strain over the time the force was applied. The stretch ratio used was $\lambda$ =$\varepsilon +1$. MATLAB was used to calculate Young’s modulus and ultimate stress and strain were ascertained as the highest values before PAC rupture.

2 $$E=\frac{\sigma }{\varepsilon }=\frac{F{L}_{0}}{A\Delta L}$$

3 $$\varepsilon =\frac{\Delta L}{{L}_{0}}$$

### Second Harmonic Generation Imaging (SHG) and immunofluorescence

**Fig. 5:**
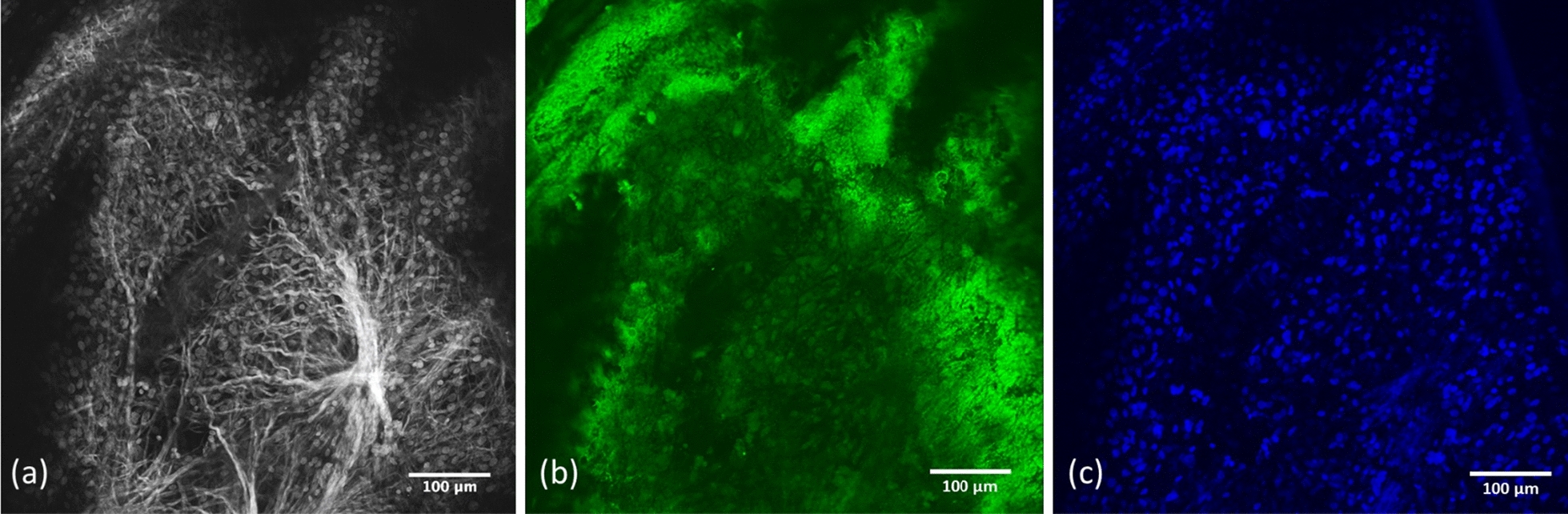
**a** Second Harmonic Generation (SHG) imaging of the PAC at 860 nm. **b** Anti-Von Willebrand Factor applied at a concentration by volume of 1:1000 to view the membrane lining of endothelial cells in blood vessels. **c** DAPI applied at concentration by volume of 1:1000 to view cell nuclei

SHG imaging was used to visualize the collagen fiber microstructure in PAC samples. Images were taken at 20 × magnification and 860 nm excitation wavelength using a confocal/multiphoton microscope (Fluoview 1000, Olympus, Valley Center, PA) (Fig. 5a). To visualize microvascular structure and cell nuclei, the samples were fixed in 4% paraformaldehyde for 24 h and washed 3 × with PBS + 0.1% Triton X-100 (PBST). The samples were then blocked with 10% bovine serum albumin in PBST for 1-h. To stain blood vessels, samples were incubated with anti-von Willebrand Factor (ab6994, Abcam, Cambridge MA) primary antibody at 1:1000 dilution ratio in 5% bovine serum albumin in PBST on a rocker overnight at 4 °C (Fig. 5b). Samples were then washed 3 × for 5 min in PBST and incubated with anti-goat Alexa Fluoro 488 secondary antibody (1:2000 dilution ratio) for 1 h at room temperature. Cell nuclei were co-stained with 4,6-diamidino-2-phenylindole (DAPI) (Invitrogen). Samples were washed 3 times for 5 min each in PBST and imaged with the confocal microscope (Fig. 5c).

**Fig. 6:**
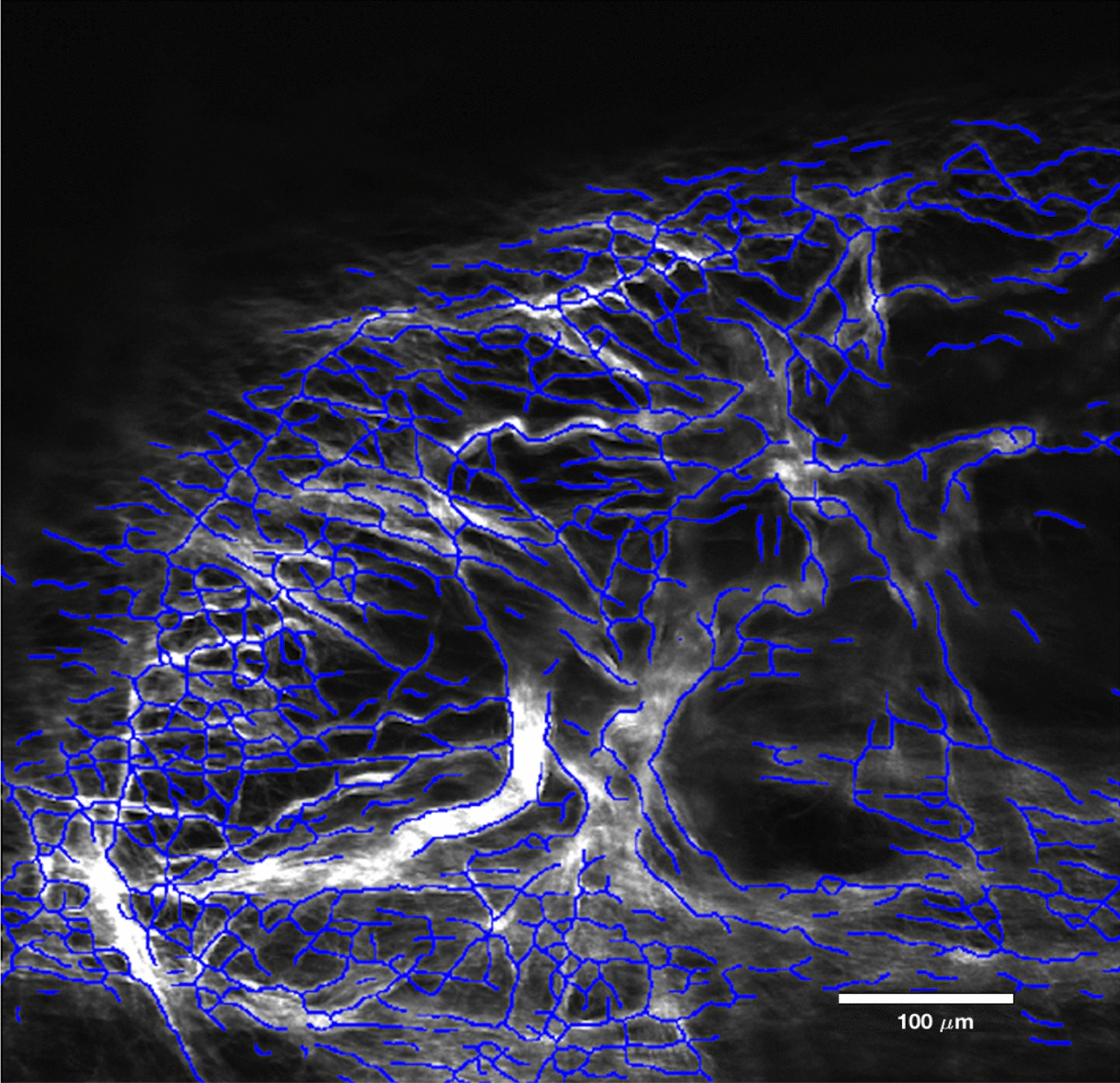
Single image of the SHG image stack from fiber analysis code developed by Koch et al. Blue lines depict fiber analysis result (skeleton) used to compute fiber-related parameters (see methods) [24]

An automated image-based collagen fiber detection method for use with multi-photon microscopy [24] was used to derive fiber orientation and structure in multiple planes (Fig. 6). Slice thickness for z-direction stacks was chosen to be 2.32 µm. This method quantified fiber intersection density, concentration, porosity, tortuosity, segment length, orientation, radial counts, and diameter. These parameters resulted in imaged samples (n = 4) having approximately 20 to 50 slices.

### Statistical analysis

Descriptive statistics were obtained for stress–strain parameters resulting from different PAC locations (occipital and frontal, and left and right hemispheres). To determine if regional differences existed between tissue sample locations, a two-sample independent t-test with unequal variance was applied using MATLAB. The parameters tested were Young’s Modulus, ultimate stress, and ultimate strain. Differences were considered significant at p-value < 0.05.

## Results

### PAC mechanical properties

Testing was performed less than five hours post-mortem with an average strain rate of 0.59 ± 0.12 s^−1^. A two-sample independent t-test applied to the occipital and frontal lobes, and left and right hemispheres showed no statistically significant differences between regions for Young’s modulus, ultimate stress, or ultimate strain (p > 0.05) (Table 1). Thus, all samples were pooled to find average length, width, thickness, Young’s modulus, ultimate stress, and ultimate strain of 4.06 ± 0.61, 3.57 ± 0.90 mm, 70. ± 30. µm, 7.7 ± 3.0, 2.7 ± 0.13 MPa, and 0.60 ± 0.14, respectively. The isotropic material parameters in the Mooney-Rivlin curve fit, C_10_, C_01_, and C_20_ (Eq. 1) were calculated to be 1, −1.004 and 0.629 MPa, respectively (SSE = 0.6552, R^2^ = 0.985), with 95% confidence bounds.

**Table 1:** Review of previously published literature documenting PAC mechanical properties and comparison of results to the present study

Ref	Tissue	Species	Young’s modulus (MPa)	Test method	Strain rate (s^−1^)	Ult. stress (MPa)	Ult. strain (%)	Extraction location
Conley et al. 2020 (present study)	PAC	Ovine	7.68 ± 3.0	Tensile	0.59	2.69 ± 0.76	0.60 ± 0.13	Brain
Jin et al. [22]	PAC	Bovine	6.75 ± 0.75	Tensile	0.05	1.05 ± 0.04	35.5 ± 3.9	Brain
Jin et al. [22]	PAC	Bovine	7.52 ± 0.59	Tensile	0.5	1.17 ± 0.13	37.1 ± 2.2	Brain
Jin et al. [22]	PAC	Bovine	10.78 ± 0.58	Tensile	5	1.53 ± 0.11	30.0 ± 3.3	Brain
Jin et al. [22]	PAC	Bovine	40.19 ± 3.54	Tensile	100	3.48 ± 0.25	21.0 ± 1.6	Brain
Kimpara et al. [25]	PAC	Porcine	26.5 ± 3.51	Tensile	0.005	9.8 ± 1.7	43.2 ± 5.6	Spinal Cord—Denticulate ligament
Kimpara et al. [25]	PAC	Porcine	30.6 ± 7.35	Tensile	0.05	10.7 ± 3.1	45.6 ± 12.7	
Kimpara et al. [25]	PAC	Porcine	63.9 ± 13.0	Tensile	0.5	20.1 ± 3.4	37.7 ± 11.0	
Kimpara et al. [25]	PAC	Porcine	21.7 ± 2.88	Tensile	0.005	6.0 ± 1.8	29.2 ± 5.6	Spinal cord—Posterior median septum
Kimpara et al. [25]	PAC	Porcine	25.4 ± 3.61	Tensile	0.05	7.2 ± 1.2	35.0 ± 4.8	
Kimpara et al. [25]	PAC	Porcine	39.3 ± 10.4	Tensile	0.5	9.7 ± 2.5	31.6 ± 6.3	
Kimpara et al. [25]	PAC	Porcine	10.8 ± 1.74	Tensile	0.005	2.1 ± 0.6	23.2 ± 5.5	Spinal Cord—Posterolateral sulcus
Kimpara et al. [25]	PAC	Porcine	12.4 ± 1.13	Tensile	0.05	2.3 ± 0.4	21.6 ± 5.1	
Kimpara et al. [25]	PAC	Porcine	13.2 ± 0.98	Tensile	0.5	2.6 ± 0.6	23.4 ± 6.5	
Ozawa et al. [26]	PAC	Rabbit	2.3	Tensile	N/A	N/A	N/A	Spinal Cord

### SHG imaging

SHG imaging allowed for visualization of collagen microstructure in terms of sheets, pillars, and blood vessel walls in a subset of PAC samples (n = 4, Fig. 5a). Anti-von Willebrand Factor and DAPI staining confirmed the presence of endothelial cells and cell nuclei throughout the PAC samples (Fig. 5b, c). This process helped differentiate SAT from transverse blood vessels. SAT fibers showed straight and crimp-like morphology (Fig. 5a and Fig. 6). Using fiber tracing and analysis software (Fig. 6), the average SAT fiber intersection density, concentration, porosity, tortuosity, segment length, orientation, radial counts, and diameter were found to be 0.23, 26.14, 73.86%, 1.07 ± 0.28, 17.33 ± 15.25 µm, 84.66 ± 49.18°, 8.15%, and 3.46 ± 1.62 µm, respectively.

## Discussion

This study quantified the mechanical properties of the PAC, with the goal of improving simulations of TBI and other CNS pathologies. The protocol for material testing and the use of the Mooney-Rivlin model was similar to previously published studies. However, to our knowledge, this study represents the first biomechanical and morphological quantification of ovine PAC tissue under uniaxial tension. Methods for brain extraction/dissection and uniaxial tension experiments were closely followed according to previous studies. Fresh ovine PAC samples (n = 10) were kept on ice and submerged in artificial CSF between dissection and testing locations. Samples were removed from ice, taken out of their respective containers and kept moist at room temperature, and tested within 5 h post-extraction. SHG imaging and fiber analysis of the collagen microstructure within the PAC indicated an isotropic collagen structure (for example shown by a wide range of variation in fiber orientation: 84.66 ± 49.18°). Taken together, these findings provide normative values for PAC material properties and structure that can be used for modeling and understanding disease states.

### Comparison of biomechanical results to previous studies

**Fig. 7:**
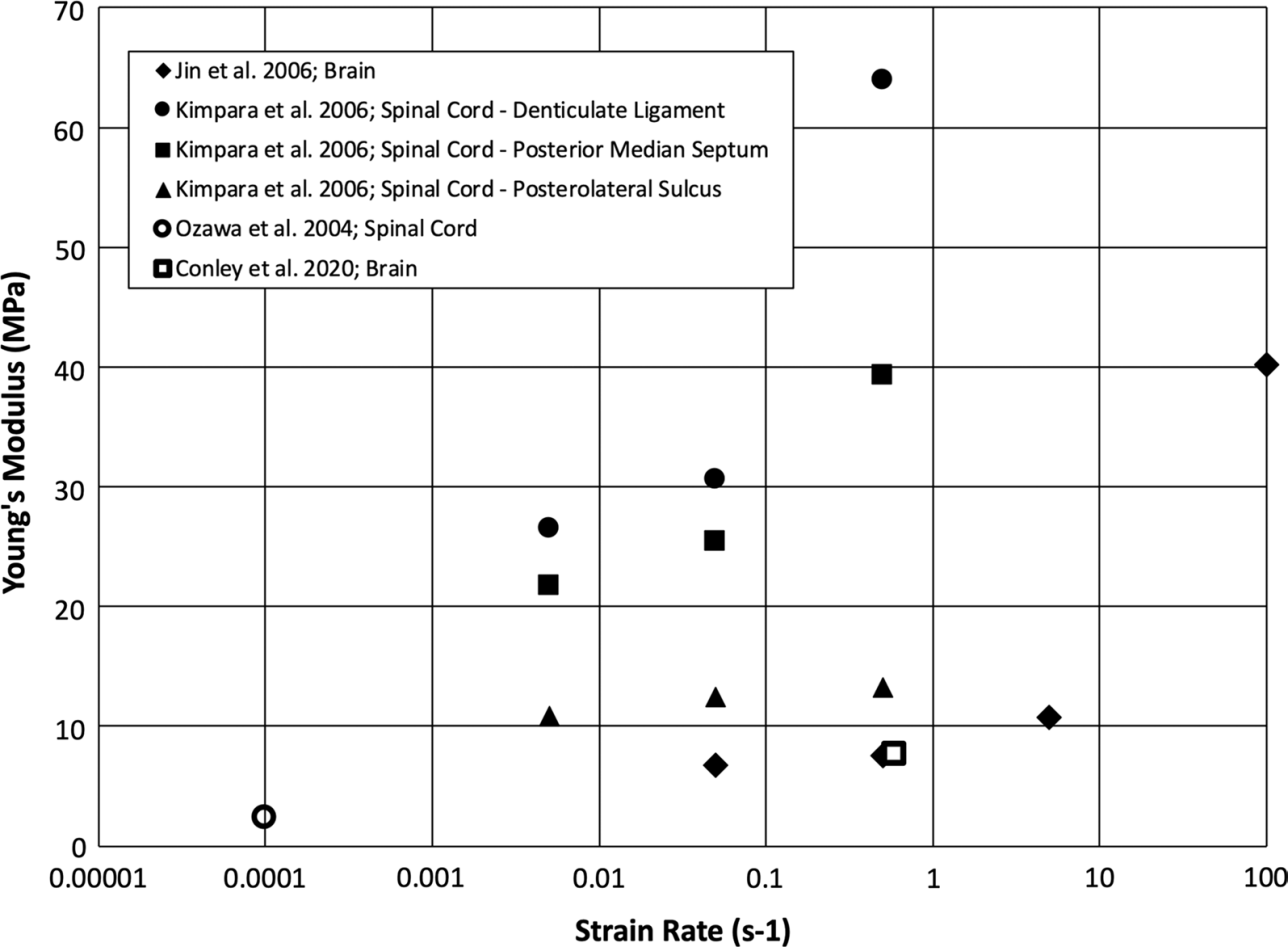
Relation of Young’s Modulus and strain rate computed for previous studies published in the literature based on isolated PAC in the spine and brain across bovine, ovine, porcine, and rabbit

PAC biomechanical properties under uniaxial tension have been found to vary from ~ 7 to 65 MPa across a range of species and testing protocols (Table 1 and Fig. 7) [22, 25, 26]. In our study, Young’s modulus was found to be at the lower range of those values at 7.7 ± 3.0 MPa. Prior work using a testing methodology most comparable to our study identified the Young’s modulus of bovine PAC to be 7.52 MPa, at a strain rate of 0.5 s^−1^ [22]. Another study in bovine spinal cord estimated the spinal PAC to be 17 MPa at a strain rate of 0.05 s^−1^ [25]. Here, biomechanical differences across the four tested regions of the brain were found to be similar, with no statistically significant differences detected in Young’s modulus, ultimate stress and strain, or thickness. These findings are consistent with prior results in bovine models [21, 25].

To our knowledge, human PAC biomechanical properties have not been documented, and therefore a direct comparison with our results is not possible. While studies using human tissues are limited, the volume fraction in post-mortem human SAT was found to be 5 to 10% greater in frontal regions of the brain compared to other regions [27]. In principle, these differences in volume fraction could lead to relatively small regional differences in mechanical properties, but these relatively small regional differences were not possible to confirm in the present study. Future work may confirm or deny the relationship between region-dependent SAT volume fraction and stress-loading during TBI.

Ours is also one of the first studies to measure brain PAC directly in “fresh” unfrozen samples. The measured PAC thickness averaged 70 ± 30 µm and was measured using a micrometer with a thimble compression spring. A prior study used a cryostat sliced sample embedded in cow cheek muscles and an optical microscope to measure average bovine brain PAC thickness as 23.6 ± 5.8 µm [22]. While existing data in brain PAC are limited, prior studies have found spinal PAC thickness to vary from roughly 10–300 µm, depending on the measurement method [21, 25, 26, 28]. Using a camera image obtained from the side of the sample, female ovine spinal cord PAC was found to have a thickness of 200 ± 40 µm [28]. Finally, the thickness of rabbit spinal cord PAC has been estimated at 12 ± 3 µm [26]. Collectively, variations in thickness across studies likely result from the different methodologies, species, and regions analyzed. These variations underscore the importance of generating unique PAC material parameters for simulations based on the location of the simulated trauma.

### PAC material model

Similar to previous studies investigating CNS tissue biomechanics [29–31] a Mooney-Rivlin model was applied to estimate material constants that were based on experimentally generated stress–strain curves. The present study found the Ovine PAC Mooney-Rivlin material parameters C_10_, C_01_, and C_20_ (Eq. 1) to be 1, −1.004 and 0.629 MPa, respectively. The parameters C_10_, C_01_, and C_20_ are material constants related to the distortional response of the tissue, which are determined according to curve-fitting accuracy requirements in this study. However, the constitutive relationship described by Eq. 1 is phenomenological. The use of a phenomenological constitutive relationship has the characteristic that its material constants do not bear a clear physiological meaning. Jin et al. also used the Mooney-Rivlin model based on Bovine PAC under uniaxial tension and found constants to be C_31_ = 1.3 (kPa), C_32_ = 56 (kPa), C_41_ = 27.5 (kPa), C_42_ = 7.9 (kPa), C_51_ = 16.55 (MPa), C_52_ = 8.45 (MPa) [32]. Other studies have used similar methods regarding the mathematical model for biological tissues [30, 33–35]. The Mooney-Rivlin model was also chosen since previous research [36] has found the relation between shear stress and shear strain to be linear for brain tissue. However, this assumption may not be correct for PAC tissue that is separate from the brain parenchyma. A constituent-based stress–strain relationship could help account for the main wall constituents and their structural properties [37], as described by the image-based fiber detection method in our study, giving thus a clear physiological meaning to the constants defining the elastic properties of the tissue.

### Structural morphology

This study utilized a novel method for SAT morphology quantification. SHG imaging with an 860 nm wavelength was used to visualize and analyze the type I collagen SAT structure. As the majority of cranial SAT are type I collagen [38], SHG is an effective tool for producing high fidelity images of the SAT structure. Additionally, using these images, SAT fiber intersection density, concentration, porosity, tortuosity, segment length, orientation, radial counts, and diameter were characterized.

SAT fiber morphology has traditionally been studied with scanning electron microscopy, transmission electron microscopy, and brightfield microscopy. Scanning electron microscopy and transmission electron microscopy showed that the structural morphology of human (post-mortem) SAT consisted of pillars, columns, sheets, branched fibrils, and other complex structures with fibril diameters that varied from 0.5–3 µm [3]. Optical coherence tomography was utilized to show that SAT fiber diameter varies from 19.2–45.5 µm [27]. Fiber diameter in the human (post-mortem) bulbar subarachnoid space was also found to be between 0.2–1 µm [39]. The present study used SHG imaging in concert with a program developed by Koch et al. [24] and found fiber diameter of ovine brain PAC to be 3.5 ± 1.6 µm. Fibril diameter variation is likely due to different species or varied anatomical sampling locations. Few studies have explored the linkage between morphology and biomechanical properties. Fabris et al., performed atomic force microscopy indentation and immunofluorescent staining on ex-vivo rat PAC and found significantly different stiffness with respect to reflecting trabecular density [40]. They also correlated stiffness with increased vascularization and vimentin density. The linkage between the image-based morphological characteristics and their specific impact on biomechanical properties should be further explored.

Biological tissues typically have a non-linear stress–strain behavior, which is characterized by a toe region at low strains. Collagen tissues have a wavy structure (crimp) that is thought to produce this behavior due to its unfolding or rotation of fibers parallel to the stretch direction.[41–43]. Based on the SHG imaging of the collagen structure in the PAC, which was performed at the zero-stretch state of the tissue, it is possible that the non-linear stress–strain behavior we observed is a result of a re-orientation of the underlying collagen fiber network along the stretch direction. Additional studies are needed to determine how stretch affects the collagen fiber orientation and structural parameters in the subarachnoid space. To further understand possible location variances in this matrix, histological analysis of the PAC and the collagen crimp structure is needed.

### Relevance of results to TBI modeling

In TBI, stress loads on the brain may be dependent on PAC and SAT morphology, similar to the significant biomechanical changes observed in spinal cord tissue properties when the PAC is removed [28]. These morphologies may change after multiple sub-concussive or concussive impacts, altering the impact stress loads on the brain from future head injuries and ultimately making the subject potentially prone to TBI. The removal or absence of pia has been shown, through simulations, to have an important effect on pressure distribution in the spine [44, 45]. The correlation between how TBI affects morphological changes and how those changes impact biomechanical properties is still largely unknown, but merits further investigation. Accurate representation of this tissue in simulations of TBI is critical for protective devices to be developed in high-risk situations such as impact sports. The provided biomechanical and morphological parameter results give information about a new animal model that can be used for finite element analysis experiments and interspecies comparisons.

### Limitations

Variability across studies may be dependent on extraction and isolation methods [46] since PAC is sensitive to temperature and moisture content, and known to degrade and change rapidly post-mortem [28]. The present study employed methods analogous to prior work [22]. To avoid potential structural alteration with the testable tissue samples, thickness measurements were averaged from a separate set of samples (n = 10).

The resting state of the PAC is pre-stressed in situ on the brain. When removed from the surface of the brain, the tissue shrinks in size to a zero-stress condition. Similar to previous studies, a zero-stress condition was assumed. In the present study, the load frame coordinates were lowered by 2 mm after the PAC sample was secured in the soft grips to eliminate pre-stress conditions. An average strain rate of 0.59 s^−1^ was applied because this was the maximum strain rate that could be delivered by our apparatus. Specimen width was found to change by as much as 63% between its pre-stressed condition situated on the brain, to its unstressed condition in the C-clamp pre-test. To remain consistent with previous studies, room temperature (22 °C) was maintained throughout uniaxial tension tests [21, 22, 47]. Dehydration was shown to increase stiffness and strength in tissues made of collagen [48]. Other biological tissues show biomechanical alterations due to dehydration including the cellular level [49–52]. Anti-drying methods include saline solution spray [53, 54], silicone adhesive coating [55], silicon oil [56], or submersion in a saline bath [57]. The present study kept the samples hydrated with artificial CSF. Hartmann et al. developed the only known technique for in-vivo human SAT visualization and reported the depths of different neural structures [58]. This technique could be modified to obtain SAT morphological parameters.

In addition to the potential of tissue degradation, this study had a relatively low sample size. This was due to the number of brains and PAC samples that experienced visible tissue damage. Future studies may develop a gentler dissection method to protect the tissues. Additionally, average sample thickness was quantified using samples from multiple areas of the brain. While tests showed no significant difference in the mechanical properties of the four sample locations, future studies could control thickness based on the area (occipital, frontal and etcetera). Finally, this study did not control for age or sex. With higher sample sizes it would be possible to determine variances that occur with age or sex in addition to location.

Importantly, this and many previous studies were carried out using animal tissues. As our work potentially indicates that mechanical properties of the PAC vary based on species, quantifying the difference in mechanical properties relative to human tissue is vital for generating appropriate mechanical parameters for simulations of human TBI. Additional work could be carried out in concussed and non-concussed human samples of individual SAT fibers. These tests could be done along multiple axis with multiple strain rates. Future studies could be also be conducted with an alternative apparatus to test for higher strain rates that would be more applicable in TBI. An increased sample size could improve statistical significance of stress strain relationship between brain location and species.

## Conclusion

This study represents the first biomechanical characterization of ovine PAC tissue. Methods were successfully developed to rapidly harvest and test samples across four extraction locations. Results are consistent and reproducible with previous studies of PAC biomechanical properties. This can be used to narrow hypotheses made about the stress–strain curve of PAC across different species and, in the future, humans. Further development of the understanding of PAC properties can be used to establish boundary conditions and nonhomogeneous material properties for numerical modeling of TBI under variable stress loads.

## Data Availability

The datasets used and/or analyzed during the current study are available from the corresponding author on reasonable request.

## Abbreviations

CSF: Cerebrospinal Fluid
CNS: Central Nervous System
DAPI: 4,6-Diamidino-2-phenylindole
PAC: Pia-Arachnoid Complex
PBS: Phosphate Buffered Saline
PBST: Phosphate Buffered Saline with Triton
SAT: Subarachnoid Trabeculae
SHG: Second Harmonic Generation
TBI: Traumatic Brain Injury
